# Proteins associated with future suicide attempts in bipolar disorder: A large-scale biomarker discovery study

**DOI:** 10.1038/s41380-022-01648-x

**Published:** 2022-06-13

**Authors:** Johan V. Sandberg, Caroline Hansson, Andreas Göteson, Erik Joas, Joel Jakobsson, Erik Pålsson, Mikael Landén

**Affiliations:** 1 Department of Psychiatry and Neurochemistry, Institute of Neuroscience and Physiology, University of Gothenburg, Gothenburg, Sweden; 2 Institute of Stress Medicine, Region Västra Götaland, Gothenburg, Sweden; 3 Department of Medical Epidemiology and Biostatistics, Karolinska Institutet, Stockholm, Sweden

**Keywords:** Predictive markers, Bipolar disorder

## Abstract

Suicide is a major cause of death worldwide. Several biological systems have been implicated in suicidal behavior but studies of candidate biomarkers have failed to produce clinically relevant biomarkers for suicide prediction. The objective of the present study was to identify novel candidate biomarkers for suicidal behavior. We used a nested case-control study design where a large cohort of patients with bipolar disorder (*N* = 5 110) were followed up to 8 years after blood sampling. We included patients that attempted suicide during follow-up (*N* = 348) and matched bipolar disorder patients from the same cohort who did not attempt suicide during the study period (*N* = 348) and analyzed a total of 92 proteins with a neuro exploratory multiplex panel. Using a multivariate classification algorithm devised to minimize bias in variable selection, we identified a parsimonious set of proteins that best discriminated bipolar disorder patients with and without prospective suicide attempts. The algorithm selected 16 proteins for the minimal-optimal classification model, which outperformed 500 models with permuted outcome (*p* = 0.0004) but had low sensitivity (53%) and specificity (64%). The candidate proteins were then entered in separate logistic regression models to calculate protein-specific associations with prospective suicide attempts. In individual analyses, three of these proteins were significantly associated with prospective suicide attempt (SCGB1A1, ANXA10, and CETN2). Most of the candidate proteins are novel to suicide research.

## Introduction

More than 800,000 people die from suicide each year [1] and an estimated 90% of suicide victims suffer from a psychiatric disorder amenable to treatment [2]. With respect to bipolar disorder, the suicide rate is 15–20 times higher than that of the general population [3, 4] and it has been estimated that 40% of persons with bipolar disorder attempt suicide during their lifetime [5]. While the risk for suicide [6] and suicide attempts [7] can be lowered by the mood stabilizer lithium, tools for suicide risk stratification and targeted anti-suicidal interventions are lacking. Indeed, a systematic review by the Swedish Agency for Health Technology Assessment and Assessment of Social Services concluded that there are no instruments with enough reliability to predict suicide or suicide attempts [8]. Further, while several risk factors for suicide and suicide attempts in bipolar disorder have been identified [9, 10], a recent meta-analysis concludes that even the top risk factors have very small effect sizes and are of limited clinical significance [11]. In fact, after over a century of suicide research the ability to predict suicide has improved little and remains near chance [11, 12].

Several biological systems have been explored in relation to suicidal behavior [13–17], but findings have not translated to clinically useful biomarkers. A meta-analysis of candidate biomarkers from the monoamine, immune, and endocrine systems found that effects were uniformly small [18] and studies of brain-derived neurotrophic factor (BDNF) have reported conflicting findings [19]. Given the consistently small effect sizes for isolated candidate biomarkers and the complex interactions between different biochemical systems [20, 21], a viable biomarker model for suicide risk might require the combination of a broader set of biomarkers where many are yet to be discovered. Indeed, a study on suicidality in bipolar disorder using a whole-genome gene expression approach found suicidality to be associated with mRNA levels of genes that had not previously been considered candidates for suicidal behavior [22].

The aim of the present study was to identify novel potential biomarkers for *future* suicide attempt through an exploratory approach focusing on candidate systems rather than specific candidate markers. Using a neuro exploratory multiplex biomarker panel, we measured a total of 92 unique proteins involved in, e.g., neurogenesis, neural plasticity, immune system processes, and stress response. We employed a nested case-control study design where a large cohort of patients with bipolar disorder (*N* = 5 110) were followed up to 8 years after blood sampling. We included the patients that attempted suicide during follow-up (henceforth denoted ‘cases’, *N* = 348) and matched bipolar disorder patients from the same cohort who did not attempt suicide during the study period (henceforth denoted ‘controls’, *N* = 348).

## Methods

### Study participants

Participants were enrolled in the Swedish Bipolar Collection (SWEBIC) study between 2009 and 2013 [23]. Patients were primarily identified through the Swedish quality register for bipolar disorders (BipoläR) [9]. BipoläR was established in 2004 and contains individualized data on bipolar subtype (bipolar type I, II, and not otherwise specified [NOS]). Diagnoses are made in regular care and diagnostic assessments reflect clinical routine. The first registration can occur at any point during the course of illness. Information is typically collected by the treating psychiatrist, or other staff trained in the diagnosis and treatment of bipolar disorder, who have access to all clinical data for the patient. Study persons were also recruited to the SWEBIC study through the Swedish National Patient Register using a validated algorithm requiring at least two hospitalizations with a BD diagnosis [24]. Study nurses conducted a confirmatory structured telephone interview including a diagnostic review.

A total of 5 110 bipolar patients were enrolled in the SWEBIC study. Blood was donated at nearest lab or hospital and drawn in 9 ml EDTA tubes (Becton, Dickinson and Company) that were sent by regular overnight mail to the Karolinska Institutet Biobank, Stockholm. Upon arrival, the samples were centrifuged for 15 min at 2000 x g, and blood plasma were separated in 0.5 ml aliquots and stored at −80 °C. Typically, the blood samples arrived at the biobank within 24 h of sample collection.

Data on suicide attempts were obtained by linking data from the SWEBIC study to the Swedish National Patient Register, which has nationwide information on psychiatric inpatient care since 1973 and outpatient visits in specialist care since 2001 [25]. Most diagnoses in the inpatient register have a positive predictive value of about 85–95% [25]. Here, suicide attempts were classified according to ICD-10 codes as either certain suicide attempt (X60-84) or suicide attempt with undetermined intent (Y10-34). The register linkage was conducted in August 2017. Hence, the follow-up time ranged from 4 to 8 years after blood sampling.

**Fig. 1:**
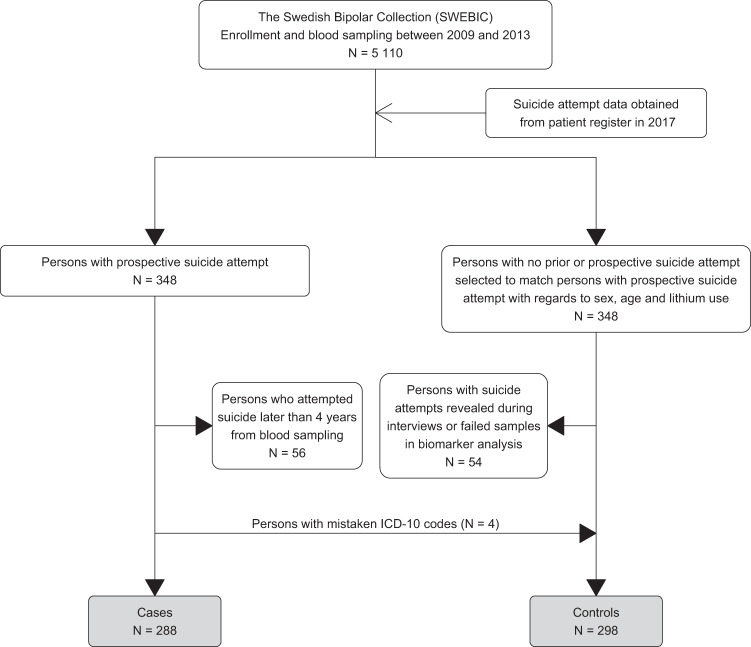
Flowchart of study person selection from SWEBIC.

Figure 1 shows a flowchart of study person inclusion. Out of the 5 110 persons with bipolar disorder who donated blood at baseline, 348 persons had attempted suicide during follow-up. Cases were matched for sex, age and lithium use with 348 controls from the same cohort of bipolar disorder patients who did not have any registered previous or prospective suicide attempts. 54 persons from this matched control group were subsequently excluded (i) due to prior suicide attempts that was not registered in the patient register but revealed during interviews (*N* = 52) or (ii) due to failed samples in the biomarker analysis (*N* = 2). In order to achieve follow-up period uniformity between cases and controls, we also excluded 56 persons whose attempted suicide occurred later than four years after blood sampling. Four persons were misclassified as cases due to mistaken ICD-10 codes and were moved to the control group. A total of 586 persons were included in the final analysis: 288 cases and 298 controls.

The study was approved by the Regional Ethical Review Board in Stockholm, Sweden, and all participants provided written and oral informed consent.

### Biomarker analysis

Protein concentrations in plasma were quantified using the Olink® Neuro Exploratory panel provided by Olink Bioscience (Olink Bioscience, Uppsala, Sweden) [26]. This multiplex panel gives a relative quantification of 92 proteins in each 96-well plate. Plasma samples from patients were randomized across wells on eight plates. Each protein is detected by a matched pair of antibodies, coupled to unique oligonucleotides, and protein concentrations are measured by a quantitative real-time polymerase chain reaction (PCR). The analyses were performed by Olink Bioscience. For 15 of the 92 proteins, more than 25% of the analyzed samples fell below the limit of detection. Those proteins were considered to have too many unreliable observations and were omitted from further analyses. The average intra- and inter-assay coefficients of variance, based on duplicate pooled plasma samples on each plate, were 7% and 11%, respectively. Data received from Olink® are presented as normalized protein expression (NPX) [26] corresponding to log2-transformations of “intensity normalized” protein values, i.e., where plate-variation was further standardized by setting an equal median per assay and plate. Proteins are referred to by encoding gene symbol. Supplementary Table 1 lists all 77 included proteins with full protein names.

### Statistical analyses

We first identified a parsimonious set of proteins associated with prospective suicide attempt by removing uninformative proteins using the MUVR algorithm [27]. This algorithm minimizes the risk of statistical overfitting by performing variable selection within a framework of repeated double cross-validation. The variable selection occurs through backward elimination in a recursive process of averaging variable ranks derived from classification models of randomly sampled segments of the data. We opted for modelling using partial least squares-discriminant analysis (PLS-DA) [28], where variables are ranked by variable importance of projection [29]. To estimate the statistical significance of the final minimal-optimal model [27], i.e., the model with the minimal set of predictors for optimal prediction performance, we conducted permutation analyses. By iteratively modelling the original data on a randomly permuted response vector we generated 500 permutation models. The actual model fit was then tested for parametric fit in a distribution of permutation model misclassifications (i.e., the null hypothesis distribution). In all analyses, the MUVR algorithm internally scaled protein values to z-scores (mean = 0, standard deviation = 1) and was run through 100 repetitions (nRep = 100) with recommended key parameter settings (nOuter = 8, nInner = 7, varRatio = 0.9).

Proteins selected in the minimal-optimal PLS-DA model were then individually included in logistic regression models to calculate the odds ratios (ORs) of attempting suicide during follow-up for each protein, adjusted for sex, age, and lithium treatment. A majority of cases had also attempted suicide prior to blood sampling (168/288). To disentangle state from trait, we calculated a second set of ORs after excluding the 168 persons who had attempted suicide prior to blood sampling.

We calculated Spearman’s rank correlation coefficients to explore the association of each protein selected in the minimal-optimal PLS-DA model with: the number of days from blood sampling to the suicide attempt (*N* = 288); the number of bipolar disorder hospital admissions during the follow-up period (*N* = 502); and the severity of bipolar disorder as rated on the Clinical Global Impression scale within four years of blood sampling (*N* = 396). Finally, Welch’s *t*-tests were performed to compare mean NPX levels between cases and controls for each of the 77 included proteins.

To compensate for multiple testing, Bonferroni correction was used in the logistic regression models and the false discovery rate (FDR) method [30] was used in the Welch’s *t*-tests.

## Results

Patient characteristics are presented in Table 1. Bipolar disorder type 2 and treatment with antidepressants was more common in suicide attempters, as was smoking, and a history of alcohol and drug abuse. Information on prescription of lithium was missing in 21 patients, who were thus excluded when calculating ORs adjusted for lithium.

**Table 1:** Baseline characteristics of the 298 patients who had never attempted suicide (controls) and 288 patients with prospective suicide attempt (cases).

	Controls *N* = 298	Cases *N* = 288	*p*-value^1^
Previous SA *N*	0	168	
Age (mean ± SD)	48 ± 15	47 ± 15	
Sex *N* (%)			0.23
Male	122 (40.9)	104 (36.1)	
Female	176 (59.1)	184 (63.9)	
Diagnosis *N* (%)			
BD1	145 (48.7)	106 (36.8)	0.004
BD2	88 (29.5)	109 (37.8)	0.033
BD-NOS	52 (17.4)	61 (21.2)	0.25
Medication *N* (%)			
Lithium	149 (51.7)	143 (51.6)	0.98
Antidepressants	86 (54.4)	130 (68.8)	0.006
Antipsychotics	105 (62.1)	146 (69.5)	0.13
Smoking *N* (%)	62 (21.7)	114 (42.1)	<0.001
History of addiction *N* (%)			
Alcohol	26 (9.8)	70 (26.9)	<0.001
Narcotics	7 (10.0)	29 (28.4)	0.004
Medical history *N* (%)			
Stroke	8 (2.8)	5 (1.8)	0.45
Encephalitis	7 (2.4)	18 (6.7)	0.016
Chronic diseases *N* (%)			
Autoimmune^2^	5 (1.7)	7 (2.6)	0.49
Cancer	14 (4.9)	12 (4.4)	0.80

*SA* Suicide attempt, *SD* Standard deviation. *BD1* Bipolar disorder type 1, *BD2* Bipolar disorder type 2, *BD-NOS* Bipolar disorder not otherwise specified.

^1^Chi-squared test.

^2^Rheumatoid arthritis, multiple sclerosis, systemic lupus erythematosus, temporal arteritis, polymyalgia rheumatica, myasthenia gravis.

**Fig. 2:**
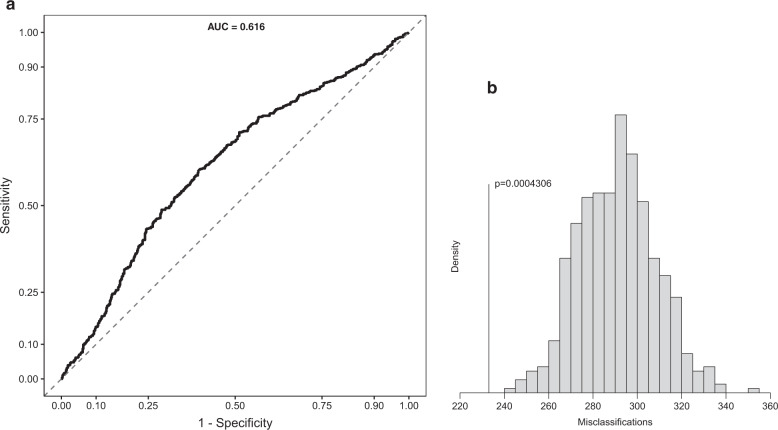
Classification model accuracy and significance. **a** ROC curve for the minimal-optimal PLS-DA model discriminating cases from control. AUC area under the curve. **b** Distribution of permutation model misclassifications. The actual model’s fit is noted with its one-tailed probability in the *t*-distribution.

The variable selection algorithm selected 16 of the 77 included proteins for the minimal-optimal PLS-DA model, henceforth referred to as *candidate proteins*. Box plots of NPX levels of the 16 candidate proteins are included in Supplementary Fig 1. The classification model outperformed all of 500 models with permuted outcome (*p* = 0.0004) as seen in Fig. 2b. However, measures of its classification performance were low (sensitivity 53%; specificity 64%; negative predictive value 59%; positive predictive value 59%). The classification accuracy of the model is summarized by the receiver operating characteristic (ROC) curve in Fig. 2a. The ranking of the candidate proteins can be seen in Table 2.

**Table 2:** Proteins are sorted by their importance for discrimination of cases from controls during variable selection. The table shows ORs of prospective suicide attempt, adjusted for age, sex, lithium prescription, with corresponding *p*-values. The primary logistic regression models include all cases and controls with non-missing values in all covariates. The secondary logistic regression models exclude 168 cases with previous suicide attempts.

		Logistic regression models					
		Primary models			Secondary models		
Protein	Rank^1^	OR	95% CI	*p*-value^2^	OR	95% CI	*p*-value^2^
SCGB1A1	1.71	0.62	[0.5, 0.8]	0.0012 *	0.93	[0.6, 1.3]	0.709
ANXA10	4.03	1.32	[1.1, 1.6]	0.0027 *	1.18	[0.9, 1.5]	0.142
CETN2	10.4	0.8	[0.7, 0.9]	0.003 *	0.77	[0.6, 0.9]	0.009
FKBP5	14.79	0.81	[0.7, 0.9]	0.006	0.81	[0.7, 1]	0.025
ATP6V1F	16.6	0.74	[0.6, 0.9]	0.007	0.73	[0.5, 1]	0.039
IFI30	17.59	1.73	[1, 3.1]	0.068	1.41	[0.6, 3.1]	0.396
IFNL1	23.47	1.34	[1, 1.7]	0.026	1.4	[1, 1.9]	0.027
BST2	23.89	1.51	[1, 2.2]	0.03	1.26	[0.8, 2]	0.345
PFDN2	24.44	0.98	[0.8, 1.3]	0.894	1.06	[0.8, 1.5]	0.732
FGFR2	26.63	1.92	[1.1, 3.4]	0.028	1.58	[0.8, 3.3]	0.213
CCL27	31.84	0.54	[0.3, 1]	0.045	0.99	[0.4, 2.2]	0.97
CD63	35.02	0.74	[0.6, 0.9]	0.014	0.77	[0.6, 1]	0.095
CRADD	35.51	0.91	[0.8, 1.1]	0.268	0.86	[0.7, 1.1]	0.164
CDH17	38.36	1.35	[1.1, 1.7]	0.018	1.23	[0.9, 1.7]	0.212
NAA10	40.05	0.87	[0.8, 1]	0.035	0.84	[0.7, 1]	0.048
PTPN1	40.26	0.9	[0.8, 1]	0.121	0.85	[0.7, 1]	0.068

*OR* Odds ratio, *CI* Confidence interval, *ANXA10* Annexin A10, *ATP6V1F* Vacuolar ATP synthase subunit F, *BST2* Bone marrow stromal antigen 2, *CCL27* C-C motif chemokine 27, *CD63* Cluster of differentiation 63, *CDH17* Cadherin-17, *CETN2* Centrin-2, *CRADD* Caspase and RIP adapter with death domain, *FGFR2* Fibroblast growth factor receptor 2, *FKBP5* PFK506-binding protein 5, *IFI30* Interferon-gamma-inducible protein 30, *IFNL1* Interferon lambda-1, *NAA10* N-alpha-acetyltransferase 10, *PFDN2* Prefoldin subunit 2, *PTPN1* Tyrosine-protein phosphatase non-receptor type 1, *SCGB1A1* Uteroglobin.

^1^Averaged ranks by variable importance of projection in the PLS-DA models created by MUVR for variable selection.

^2^α_Bonferroni_ = 0.05/16 = 0.0031.

**Fig. 3:**
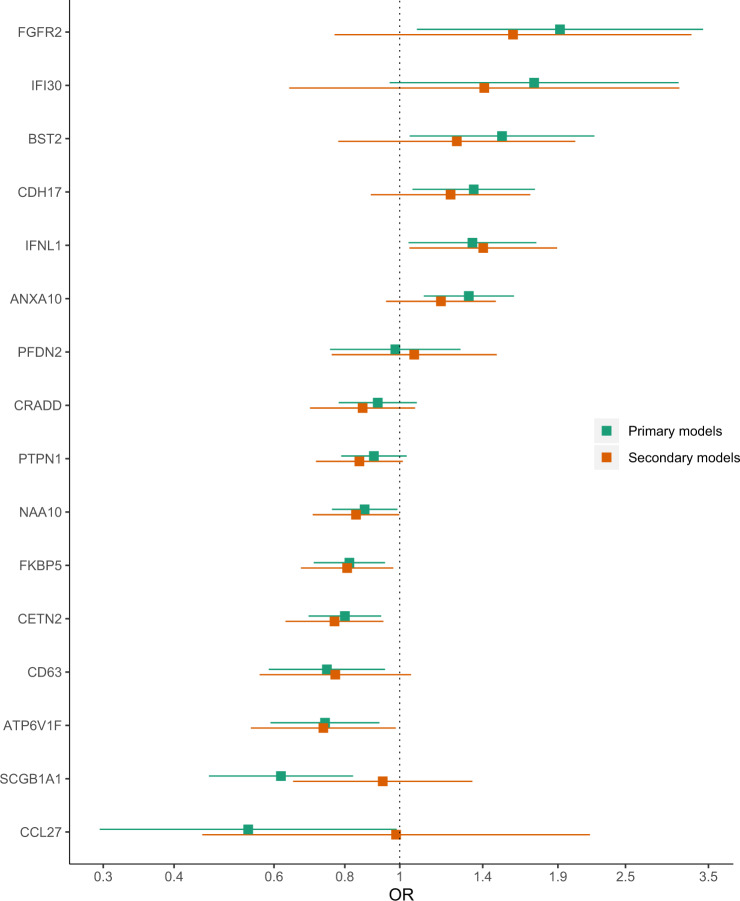
Odds ratios (ORs) for prospective suicide attempts with 95% confidence intervals for proteins from logistic regression models, adjusted for age, sex, and lithium medication. The primary models include all cases and controls with non-missing values in all covariates. The secondary models exclude 168 cases with previous suicide attempts.

In the logistic regression models, three (SCGB1A1, ANXA10, and CETN2) of the 16 candidate proteins were significantly (*p* < 0.05/16) associated with prospective suicide attempt after adjusting for sex, age, and lithium prescription (Table 2). Figure 3 shows that proteins with the largest effect sizes in the primary set of models showed smaller effect sizes in the secondary set of models, which excluded 168 cases with suicide attempts prior to blood sampling (Table 2). No protein survived correction for multiple testing in the secondary set of models.

No candidate protein plasma concentrations were significantly correlated with the number of bipolar disorder hospital admissions, Clinical Global Impression scale ratings, or the number of days that elapsed from blood sampling to suicide attempt (Supplementary Table 2).

Results from the Welch’s *t*-tests are presented in Supplementary Table 1. Two proteins (SCGB1A1 and ANXA10) differed significantly between cases and controls after correction for multiple testing.

## Discussion

We conducted an exploratory biomarker study aimed at discovering novel candidate biomarkers to predict suicidal behavior. We sampled blood plasma from 5 110 patients with bipolar disorder—a high-risk condition for suicide—and followed them prospectively for four years in the Swedish National Patient Register. We compared baseline protein concentrations of 92 proteins from 288 patients with a prospective suicide attempt during follow-up (cases) with 298 patients from the same cohort who had never attempted suicide (controls). Using PLS-DA modelling within repeated double cross-validation, we identified a parsimonious set of 16 candidate proteins associated with prospective suicide attempts (AUC = 0.616, *p* = 0.0004). Of the 16 candidate proteins, three (SCGB1A1, ANXA10, and CETN2) were significantly associated with prospective suicide attempt in individual logistic regression analyses. When patients with previous suicide attempts were excluded, no associations survived correction for multiple comparisons and many proteins showed smaller effect sizes. There were no correlations between candidate proteins and the number of days that elapsed from blood sampling to suicide attempt. In addition, we found no significant correlation of candidate proteins with disease severity or the number of bipolar disorder hospital admissions.

The observed effect sizes were small with ORs for prospective suicide attempt ranging from 0.5 to 2 for log2-transformed protein values. The small effect sizes echo previous research on suicide biomarkers [18] as well as risk factors for suicide in general [11]. Thus, the biomarker potential of the individual proteins is limited but they could be candidates for inclusion in future studies investigating multivariate prediction models for suicide risk or the neurobiology of suicidal behavior.

Four of our 16 candidate proteins have previously been investigated in relation to mood disorders or suicide (FKBP5, FGFR2, SCGB1A1, and CD63), but most candidate proteins are novel to mood disorders and suicide research.

Uteroglobin (SCGB1A1), also known as clara cell protein, was the highest ranked protein in the multivariate model and showed the second-most decreased OR for prospective suicide attempt. Low SCGB1A1 concentrations have previously been observed in serum of patients with depression [31, 32], in response to stress in patients with stress-induced anxiety [33], and in plasma of patients with schizophrenia [34]. SCGB1A1 suppresses cytokine secretion [35] and also inhibits phospholipase A2 activity [36]. Phospholipase A2, in turn, has been studied extensively in relation to psychiatric disorders [37] and may be involved in the mechanism of action of mood stabilizers [38].

Annexin A10 (ANXA10), ranked second in the multivariate model, is novel to psychiatric research. The annexins are a family of calcium-dependent phospholipid binding proteins implicated in pathologies such as cancer, cardiovascular disease, and inflammation [39]. Several annexins have also been shown to inhibit phospholipase A2 [40], mentioned above.

Centrin-2 (CETN2), ranked third in the multivariate model, is a calcium-binding component of the centrosome. It is required for duplication of centrioles during cell division [41] and also participates in ciliogenesis [42] and nucleotide excision repair [43]. Expression of CETN2 has previously been found to be down-regulated in the striatum of depressed subjects [44].

FK506-binding protein 5 (FKBP5), ranked fourth in the multivariate model, is a protein that might play a role in the dysregulation of hypothalamic–pituitary–adrenal (HPA) axis activity observed in affective disorders. It inhibits glucocorticoid receptor sensitivity [45] and has been implicated in bipolar disorder [46], depression [47] and posttraumatic stress disorder [48]. Importantly, gene expression and protein expression of FKBP5 is reduced in the amygdala of suicide victims [49], and polymorphisms in this gene has been associated with suicide as well as suicide attempts [50–52].

Fibroblast growth factor receptor 2 (FGFR2) showed the most increased OR for prospective suicide attempt. FGFR2 belongs to the fibroblast growth factor family, which is a widely researched system that has been associated with depression [53] as well as stress response and anxiety [54]. Decreased expression of FGFR2 has been found in brain regions of postmortem depressed patients [55, 56] as well as that of suicide victims [57].

Of the six candidate proteins associated with an increased risk for prospective suicide attempt, three are involved in immune system processes (IFI30, IFNL1, and BST2). In contrast, CD63 and CCL27, involved in immune cell migration, were associated with a decreased risk for prospective suicide attempt. Low CCL27 serum concentrations have previously been associated with higher anxiety scale scores [58], which is a risk factor for suicide [9].

The remaining candidate proteins (i.e., not discussed above) are involved in, e.g., cell cycle-related processes (CRADD, and CDH17), cell migration (NAA10), chaperone activity (PFDN2), and metabolism (PTPN1 and ATP6V1F).

### Strengths and limitations

A major strength of this study is the prospective design. More than 5000 blood samples were collected from patients who were then followed for four years to capture suicide attempts. An additional strength is that both cases and controls were diagnosed with bipolar disorder, which is important to disentangle biological mechanisms implicated in suicidal behavior from those related to mood disorders. A limitation is that psychiatric medications other than lithium were not adjusted for in the logistic regression analyses, due to the large number of missing data on prescription of antidepressants and antipsychotics. Further, blood samples were sent to the biobank by regular mail leading to a delay between sampling and centrifugation. While most assays are resilient to factors of pre-analytical processing, pre-centrifugation delay can heavily impact certain assays [59]. However, as there was no systematic difference in pre-centrifugation delay between cases and controls, this is more likely to introduce noise than increase the risk for type I error. Finally, although candidate proteins were identified within a cross-validation framework to minimize the risk of selection bias, confirming the external validity of our findings through replication in independent cohorts is warranted.

## Conclusions

We explored a panel of 92 proteins in blood plasma from a large cohort of patients with bipolar disorder to discover novel potential biomarkers for future suicide attempts. We identified a set of 16 biomarker candidates involved in, e.g., responses to hormonal and steroid activity, immune system processes, cellular growth and metabolism. Most of our findings are novel to research on mood disorders and suicide and provide insight into the biological underpinnings of suicidal behavior. While observed effect sizes were generally small, these candidate proteins could feature in future biomarker studies evaluating the viability of combinations of candidate biomarkers as prediction models for suicide risk.

## Supplementary information

Supplementary Material

Supplementary Figure 1
