# Supplementary material for: Proteins associated with future suicide attempts in bipolar disorder: A large-scale biomarker discovery study
Source: Mol Psychiatry. 2022 Jun 13;27(9):3857–63. doi: 10.1038/s41380-022-01648-x (PMC9708594; doi:10.1038/s41380-022-01648-x)
Supplement: Supplementary file 1 — Supplementary Material [file 41380_2022_1648_MOESM1_ESM.docx]

**SUPPLEMENTARY FIGURE LEGENDS**

Supplementary Figure 1. Box plots of NPX levels of the 16 candidate proteins. 0=controls. 1=cases.

**SUPPLEMENTARY TABLES**

Supplementary Table 1. All 77 included proteins with protein names, mean differences in NPX levels between cases and controls, and results from Welsh's *t*-tests.

| Assay | Mean Difference | *t* | *p*-value | FDR adjusted *p*-value | Protein Names |
| --- | --- | --- | --- | --- | --- |
| AARSD1 | -0.08 | 1.22 | 0.222 | 0.491 | Alanyl-tRNA editing protein Aarsd1 (Alanyl-tRNA synthetase domain-containing protein 1) |
| ABHD14B | -0.05 | 0.88 | 0.379 | 0.688 | Protein ABHD14B (EC 3.-.-.-) (Alpha/beta hydrolase domain-containing protein 14B) (Abhydrolase domain-containing protein 14B) (CCG1-interacting factor B) |
| ADAM15 | 0.02 | -0.75 | 0.453 | 0.753 | Disintegrin and metalloproteinase domain-containing protein 15 (ADAM 15) (EC 3.4.24.-) (Metalloprotease RGD disintegrin protein) (Metalloproteinase-like, disintegrin-like, and cysteine-rich protein 15) (MDC-15) (Metargidin) |
| AKT1S1 | -0.1 | 1.34 | 0.18 | 0.491 | Proline-rich AKT1 substrate 1 (40 kDa proline-rich AKT substrate) |
| ANXA10 | 0.25 | -3.24 | 0.001 | 0.05 | Annexin A10 (Annexin-10) (Annexin-14) |
| AOC1 | 0.05 | -0.7 | 0.483 | 0.753 | Amiloride-sensitive amine oxidase [copper-containing] (DAO) (Diamine oxidase) (EC 1.4.3.22) (Amiloride-binding protein 1) (Amine oxidase copper domain-containing protein 1) (Histaminase) (Kidney amine oxidase) (KAO) |
| ASGR1 | 0.05 | -1.43 | 0.153 | 0.491 | Asialoglycoprotein receptor 1 (ASGP-R 1) (ASGPR 1) (C-type lectin domain family 4 member H1) (Hepatic lectin H1) (HL-1) |
| ATP6V1F | -0.17 | 2.67 | 0.008 | 0.203 | V-type proton ATPase subunit F (V-ATPase subunit F) (V-ATPase 14 kDa subunit) (Vacuolar proton pump subunit F) |
| BST2 | 0.07 | -1.89 | 0.059 | 0.306 | Bone marrow stromal antigen 2 (BST-2) (HM1.24 antigen) (Tetherin) (CD antigen CD317) |
| CARHSP1 | 0.01 | -0.22 | 0.83 | 0.952 | Calcium-regulated heat-stable protein 1 (Calcium-regulated heat-stable protein of 24 kDa) (CRHSP-24) |
| CCL27 | -0.05 | 2.01 | 0.045 | 0.3 | C-C motif chemokine 27 (CC chemokine ILC) (Cutaneous T-cell-attracting chemokine) (CTACK) (ESkine) (IL-11 R-alpha-locus chemokine) (Skinkine) (Small-inducible cytokine A27) |
| CD302 | 0.03 | -0.73 | 0.469 | 0.753 | CD302 antigen (C-type lectin BIMLEC) (C-type lectin domain family 13 member A) (DEC205-associated C-type lectin 1) (Type I transmembrane C-type lectin receptor DCL-1) (CD antigen CD302) |
| CD33 | -0.04 | 0.48 | 0.635 | 0.824 | Myeloid cell surface antigen CD33 (Sialic acid-binding Ig-like lectin 3) (Siglec-3) (gp67) (CD antigen CD33) |
| CD63 | -0.14 | 2.29 | 0.023 | 0.24 | Cluster of differentiation 63 (CD63 antigen) (Granulophysin) (Lysosomal-associated membrane protein 3) (LAMP-3) (Melanoma-associated antigen ME491) (OMA81H) (Ocular melanoma-associated antigen) (Tetraspanin-30) (Tspan-30) (CD antigen CD63) |
| CDH15 | -0.04 | 0.55 | 0.582 | 0.811 | Cadherin-15 (Cadherin-14) (Muscle cadherin) (M-cadherin) |
| CDH17 | 0.12 | -2.17 | 0.03 | 0.261 | Cadherin-17 (Intestinal peptide-associated transporter HPT-1) (Liver-intestine cadherin) (LI-cadherin) |
| CETN2 | -0.25 | 2.57 | 0.01 | 0.203 | Centrin-2 (Caltractin isoform 1) |
| CLSTN1 | -0.04 | 1.37 | 0.17 | 0.491 | Calsyntenin-1 (Alcadein-alpha) (Alc-alpha) (Alzheimer-related cadherin-like protein) (Non-classical cadherin XB31alpha) [Cleaved into: Soluble Alc-alpha (SAlc-alpha), CTF1-alpha (C-terminal fragment 1-alpha)] |
| COL4A3BP | -0.08 | 0.99 | 0.322 | 0.613 | Ceramide transfer protein (hCERT) (Collagen type IV alpha-3-binding protein) (Goodpasture antigen-binding protein) (GPBP) (START domain-containing protein 11) (StARD11) (StAR-related lipid transfer protein 11) |
| CRADD | -0.06 | 0.67 | 0.502 | 0.753 | Death domain-containing protein CRADD (Caspase and RIP adapter with death domain) (RIP-associated protein with a death domain) |
| CRIP2 | 0 | 0.03 | 0.975 | 0.99 | Cysteine-rich protein 2 (CRP-2) (Protein ESP1) |
| DEFB4A | 0.2 | -1.8 | 0.072 | 0.343 | Beta-defensin 4A (Beta-defensin 2) (BD-2) (hBD-2) (Defensin, beta 2) (Skin-antimicrobial peptide 1) (SAP1) |
| DPEP1 | -0.05 | 1.21 | 0.225 | 0.491 | Dipeptidase 1 (EC 3.4.13.19) (Beta-lactamase) (EC 3.5.2.6) (Dehydropeptidase-I) (Microsomal dipeptidase) (Renal dipeptidase) (hRDP) |
| DPEP2 | -0.01 | 0.24 | 0.807 | 0.939 | Dipeptidase 2 (EC 3.4.13.19) |
| DSG3 | -0.02 | 0.6 | 0.549 | 0.797 | Desmoglein-3 (130 kDa pemphigus vulgaris antigen) (PVA) (Cadherin family member 6) |
| ECE1 | -0.04 | 0.9 | 0.366 | 0.68 | Endothelin-converting enzyme 1 (ECE-1) (EC 3.4.24.71) |
| EIF4B | -0.07 | 1.49 | 0.137 | 0.491 | Eukaryotic translation initiation factor 4B (eIF-4B) |
| EREG | -0.12 | 1.29 | 0.198 | 0.491 | Proepiregulin [Cleaved into: Epiregulin (EPR)] |
| FCAR | 0.03 | -0.68 | 0.497 | 0.753 | Immunoglobulin alpha Fc receptor (IgA Fc receptor) (CD antigen CD89) |
| FGFR2 | 0.05 | -1.96 | 0.05 | 0.3 | Fibroblast growth factor receptor 2 (FGFR-2) (EC 2.7.10.1) (K-sam) (KGFR) (Keratinocyte growth factor receptor) (CD antigen CD332) |
| FHIT | -0.05 | 0.69 | 0.491 | 0.753 | Bis(5'-adenosyl)-triphosphatase (EC 3.6.1.29) (AP3A hydrolase) (AP3Aase) (Diadenosine 5',5'''-P1,P3-triphosphate hydrolase) (Dinucleosidetriphosphatase) (Fragile histidine triad protein) |
| FKBP5 | -0.23 | 2.35 | 0.019 | 0.24 | Peptidyl-prolyl cis-trans isomerase FKBP5 (PPIase FKBP5) (EC 5.2.1.8) (51 kDa FK506-binding protein) (51 kDa FKBP) (FKBP-51) (54 kDa progesterone receptor-associated immunophilin) (Androgen-regulated protein 6) (FF1 antigen) (FK506-binding protein 5) (FKBP-5) (FKBP54) (p54) (HSP90-binding immunophilin) (Rotamase) |
| FKBP7 | -0.06 | 1.11 | 0.266 | 0.546 | Peptidyl-prolyl cis-trans isomerase FKBP7 (PPIase FKBP7) (EC 5.2.1.8) (23 kDa FK506-binding protein) (23 kDa FKBP) (FKBP-23) (FK506-binding protein 7) (FKBP-7) (Rotamase) |
| FOLR2 | 0 | 0.1 | 0.918 | 0.989 | Folate receptor beta (FR-beta) (Folate receptor 2) (Folate receptor, fetal/placental) (Placental folate-binding protein) (FBP) |
| FUT8 | 0.02 | -0.37 | 0.711 | 0.873 | Alpha-(1,6)-fucosyltransferase (Alpha1-6FucT) (EC 2.4.1.68) (Fucosyltransferase 8) (GDP-L-Fuc:N-acetyl-beta-D-glucosaminide alpha1,6-fucosyltransferase) (GDP-fucose--glycoprotein fucosyltransferase) (Glycoprotein 6-alpha-L-fucosyltransferase) |
| GGT5 | 0.05 | -1.71 | 0.088 | 0.361 | Glutathione hydrolase 5 proenzyme (EC 3.4.19.13) (Gamma-glutamyl transpeptidase-related enzyme) (GGT-rel) (Gamma-glutamyltransferase 5) (GGT 5) (EC 2.3.2.2) (Gamma-glutamyltransferase-like activity 1) (Gamma-glutamyltranspeptidase 5) (Leukotriene-C4 hydrolase) (EC 3.4.19.14) [Cleaved into: Glutathione hydrolase 5 heavy chain, Glutathione hydrolase 5 light chain] |
| GPNMB | 0.03 | -1.28 | 0.203 | 0.491 | Transmembrane glycoprotein NMB (Hematopoietic growth factor inducible neurokinin-1 type) |
| HMOX2 | -0.11 | 1.26 | 0.209 | 0.491 | Heme oxygenase 2 (HO-2) (EC 1.14.14.18) |
| IFI30 | 0.04 | -1.79 | 0.075 | 0.343 | Interferon-gamma-inducible protein 30 (Gamma-interferon-inducible lysosomal thiol reductase) (Gamma-interferon-inducible protein IP-30) (Legumaturain) |
| IFNL1 | 0.12 | -2.3 | 0.022 | 0.24 | Interferon lambda-1 (IFN-lambda-1) (Cytokine Zcyto21) (Interleukin-29) (IL-29) |
| IL15 | 0 | 0.11 | 0.915 | 0.989 | Interleukin-15 (IL-15) |
| IL32 | 0 | -0.11 | 0.914 | 0.989 | Interleukin-32 (IL-32) (Natural killer cells protein 4) (Tumor necrosis factor alpha-inducing factor) |
| IL3RA | 0.03 | -1.39 | 0.165 | 0.491 | Interleukin-3 receptor subunit alpha (IL-3 receptor subunit alpha) (IL-3R subunit alpha) (IL-3R-alpha) (IL-3RA) (CD antigen CD123) |
| ILKAP | -0.02 | 0.18 | 0.857 | 0.969 | Integrin-linked kinase-associated serine/threonine phosphatase 2C (ILKAP) (EC 3.1.3.16) |
| ISLR2 | 0.02 | -0.45 | 0.655 | 0.824 | Immunoglobulin superfamily containing leucine-rich repeat protein 2 (Leucine-rich repeat domain and immunoglobulin domain-containing axon extension protein) |
| KIF1BP | -0.18 | 1.91 | 0.057 | 0.306 | KIF-binding protein (KIF1-binding protein) (Kinesin family binding protein) |
| KIR2DL3 | 0.05 | -0.86 | 0.39 | 0.691 | Killer cell immunoglobulin-like receptor 2DL3 (CD158 antigen-like family member B2) (KIR-023GB) (Killer inhibitory receptor cl 2-3) (MHC class I NK cell receptor) (NKAT2a) (NKAT2b) (Natural killer-associated transcript 2) (NKAT-2) (p58 natural killer cell receptor clone CL-6) (p58 NK receptor CL-6) (p58.2 MHC class-I-specific NK receptor) (CD antigen CD158b2) |
| KIRREL2 | 0.02 | -0.58 | 0.562 | 0.797 | Kin of IRRE-like protein 2 (Kin of irregular chiasm-like protein 2) (Nephrin-like protein 3) |
| KLB | 0.04 | -0.68 | 0.495 | 0.753 | Beta-klotho (BKL) (BetaKlotho) (Klotho beta-like protein) |
| LEPR | 0.03 | -1.21 | 0.227 | 0.491 | Leptin receptor (LEP-R) (HuB219) (OB receptor) (OB-R) (CD antigen CD295) |
| MAD1L1 | -0.24 | 1.98 | 0.049 | 0.3 | Mitotic spindle assembly checkpoint protein MAD1 (Mitotic arrest deficient 1-like protein 1) (MAD1-like protein 1) (Mitotic checkpoint MAD1 protein homolog) (HsMAD1) (hMAD1) (Tax-binding protein 181) |
| NAA10 | -0.17 | 1.67 | 0.096 | 0.375 | N-alpha-acetyltransferase 10 (EC 2.3.1.255) (N-terminal acetyltransferase complex ARD1 subunit homolog A) (hARD1) (NatA catalytic subunit Naa10) |
| NDRG1 | 0.03 | -0.49 | 0.627 | 0.824 | Protein NDRG1 (Differentiation-related gene 1 protein) (DRG-1) (N-myc downstream-regulated gene 1 protein) (Nickel-specific induction protein Cap43) (Reducing agents and tunicamycin-responsive protein) (RTP) (Rit42) |
| NEFL | 0.04 | -0.7 | 0.487 | 0.753 | Neurofilament light polypeptide (NF-L) (68 kDa neurofilament protein) (Neurofilament triplet L protein) |
| NPM1 | -0.1 | 1.06 | 0.289 | 0.578 | Nucleophosmin (NPM) (Nucleolar phosphoprotein B23) (Nucleolar protein NO38) (Numatrin) |
| PFDN2 | 0.03 | -0.52 | 0.604 | 0.812 | Prefoldin subunit 2 |
| PHOSPHO1 | 0 | -0.04 | 0.968 | 0.99 | Phosphoethanolamine/phosphocholine phosphatase (EC 3.1.3.75) |
| PLA2G10 | 0.03 | -0.45 | 0.65 | 0.824 | Group 10 secretory phospholipase A2 (EC 3.1.1.4) (Group X secretory phospholipase A2) (GX sPLA2) (sPLA2-X) (Phosphatidylcholine 2-acylhydrolase 10) |
| PMVK | -0.11 | 1.25 | 0.212 | 0.491 | Phosphomevalonate kinase (PMKase) (hPMK) (EC 2.7.4.2) |
| PPP3R1 | -0.19 | 2.25 | 0.025 | 0.24 | Calcineurin subunit B type 1 (Protein phosphatase 2B regulatory subunit 1) (Protein phosphatase 3 regulatory subunit B alpha isoform 1) |
| PRTFDC1 | -0.24 | 2.05 | 0.041 | 0.3 | Phosphoribosyltransferase domain-containing protein 1 |
| PSG1 | 0.03 | -0.25 | 0.802 | 0.939 | Pregnancy-specific beta-1-glycoprotein 1 (PS-beta-G-1) (PSBG-1) (Pregnancy-specific glycoprotein 1) (CD66 antigen-like family member F) (Fetal liver non-specific cross-reactive antigen 1/2) (FL-NCA-1/2) (PSG95) (Pregnancy-specific beta-1 glycoprotein C/D) (PS-beta-C/D) (CD antigen CD66f) |
| PSME1 | -0.08 | 1.32 | 0.187 | 0.491 | Proteasome activator complex subunit 1 (11S regulator complex subunit alpha) (REG-alpha) (Activator of multicatalytic protease subunit 1) (Interferon gamma up-regulated I-5111 protein) (IGUP I-5111) (Proteasome activator 28 subunit alpha) (PA28a) (PA28alpha) |
| PTPN1 | -0.11 | 1.03 | 0.305 | 0.595 | Tyrosine-protein phosphatase non-receptor type 1 (EC 3.1.3.48) (Protein-tyrosine phosphatase 1B) (PTP-1B) |
| PTS | 0.03 | -0.53 | 0.597 | 0.812 | 6-pyruvoyl tetrahydrobiopterin synthase (PTP synthase) (PTPS) (EC 4.2.3.12) |
| RBKS | 0.03 | -0.35 | 0.727 | 0.873 | Ribokinase (RK) (EC 2.7.1.15) |
| RNF31 | -0.11 | 1.26 | 0.209 | 0.491 | E3 ubiquitin-protein ligase RNF31 (EC 2.3.2.31) (HOIL-1-interacting protein) (HOIP) (RING finger protein 31) (RING-type E3 ubiquitin transferase RNF31) (Zinc in-between-RING-finger ubiquitin-associated domain protein) |
| RPS6KB1 | -0.01 | 0.08 | 0.938 | 0.989 | Ribosomal protein S6 kinase beta-1 (S6K-beta-1) (S6K1) (EC 2.7.11.1) (70 kDa ribosomal protein S6 kinase 1) (P70S6K1) (p70-S6K 1) (Ribosomal protein S6 kinase I) (Serine/threonine-protein kinase 14A) (p70 ribosomal S6 kinase alpha) (p70 S6 kinase alpha) (p70 S6K-alpha) (p70 S6KA) |
| SCGB1A1 | -0.18 | 3.52 | 0.0005 | 0.036 | Uteroglobin (Clara cell phospholipid-binding protein) (CCPBP) (Clara cells 10 kDa secretory protein) (CC10) (Clara-cell 16 kD protein) (CC16) (Secretoglobin family 1A member 1) (Urinary protein 1) (UP-1) (UP1) (Urine protein 1) |
| SFRP1 | 0.03 | -0.59 | 0.557 | 0.797 | Secreted frizzled-related protein 1 (FRP-1) (sFRP-1) (Secreted apoptosis-related protein 2) (SARP-2) |
| SMOC1 | 0 | 0.01 | 0.988 | 0.99 | SPARC-related modular calcium-binding protein 1 (Secreted modular calcium-binding protein 1) (SMOC-1) |
| SNCG | 0.08 | -1.19 | 0.236 | 0.499 | Gamma-synuclein (Breast cancer-specific gene 1 protein) (Persyn) (Synoretin) (SR) |
| SRP14 | 0.01 | -0.09 | 0.929 | 0.989 | Signal recognition particle 14 kDa protein (SRP14) (18 kDa Alu RNA-binding protein) |
| TBCB | -0.13 | 1.21 | 0.226 | 0.491 | Tubulin-folding cofactor B (Cytoskeleton-associated protein 1) (Cytoskeleton-associated protein CKAPI) (Tubulin-specific chaperone B) |
| TPPP3 | 0 | -0.01 | 0.99 | 0.99 | Tubulin polymerization-promoting protein family member 3 (TPPP/p20) |
| VSTM1 | 0.02 | -0.36 | 0.72 | 0.873 | V-set and transmembrane domain-containing protein 1 (Signal inhibitory receptor on leukocytes-1) (SIRL-1) |
| WWP2 | -0.11 | 1.44 | 0.15 | 0.491 | NEDD4-like E3 ubiquitin-protein ligase WWP2 (EC 2.3.2.26) (Atrophin-1-interacting protein 2) (AIP2) (HECT-type E3 ubiquitin transferase WWP2) (WW domain-containing protein 2) |

Supplementary Table 2. Spearman correlations of candidate proteins with: a) the number of days from blood sampling to the suicide attempt in cases (N=288); b) Clinical Global Impression (CGI) scale ratings within four years of blood sampling (N=396); c) the number of bipolar disorder hospital admissions during follow-up (N=502).

|  | a) Days to suicide attempt | | b) CGI scale ratings | | c) Hospital admissions | |
| --- | --- | --- | --- | --- | --- | --- |
| Protein | ρ^1^ | *p*-value | ρ^1^ | *p*-value | ρ^1^ | *p*-value |
| ANXA10 | 0.066 | 0.264 | 0.079 | 0.115 | -0.02 | 0.66 |
| ATP6V1F | 0.034 | 0.567 | 0.027 | 0.587 | -0.082 | 0.066 |
| BST2 | 0.009 | 0.886 | 0.029 | 0.559 | -0.052 | 0.242 |
| CCL27 | -0.031 | 0.606 | -0.093 | 0.065 | -0.023 | 0.607 |
| CD63 | 0.012 | 0.836 | -0.036 | 0.474 | -0.047 | 0.298 |
| CDH17 | 0.014 | 0.813 | -0.04 | 0.432 | -0.003 | 0.942 |
| CETN2 | -0.007 | 0.901 | 0.029 | 0.565 | -0.065 | 0.149 |
| CRADD | -0.03 | 0.607 | 0.051 | 0.316 | 0.009 | 0.841 |
| FGFR2 | 0.02 | 0.733 | 0.012 | 0.808 | 0.012 | 0.786 |
| FKBP5 | -0.033 | 0.573 | 0.053 | 0.296 | -0.044 | 0.323 |
| IFI30 | -0.013 | 0.823 | 0.023 | 0.647 | -0.027 | 0.544 |
| IFNL1 | -0.044 | 0.462 | 0.098 | 0.053 | 0.014 | 0.76 |
| NAA10 | -0.031 | 0.605 | 0.057 | 0.254 | -0.024 | 0.596 |
| PFDN2 | -0.047 | 0.431 | 0.072 | 0.15 | -0.019 | 0.664 |
| PTPN1 | -0.023 | 0.692 | 0.075 | 0.136 | -0.008 | 0.856 |
| SCGB1A1 | 0.057 | 0.339 | -0.095 | 0.059 | -0.054 | 0.225 |

ANXA10=Annexin A10. ATP6V1F=Vacuolar ATP synthase subunit F. BST2=Bone marrow stromal antigen 2. CCL27=C-C motif chemokine 27. CD63=Cluster of differentiation 63. CDH17=Cadherin-17. CETN2=Centrin-2. CRADD=Caspase and RIP adapter with death domain. FGFR2=Fibroblast growth factor receptor 2. FKBP5=FK506-binding protein 5. IFI30=Interferon-gamma-inducible protein 30. IFNL1=Interferon lambda-1. NAA10=N-alpha-acetyltransferase 10. PFDN2=Prefoldin subunit 2. PTPN1=Tyrosine-protein phosphatase non-receptor type 1. SCGB1A1=Uteroglobin.

^1^ Spearman's rank correlation coefficient.
