# Supplementary figures and images for: Proteins associated with future suicide attempts in bipolar disorder: A large-scale biomarker discovery study
Source: Mol Psychiatry. 2022 Jun 13;27(9):3857–63. doi: 10.1038/s41380-022-01648-x (PMC9708594; doi:10.1038/s41380-022-01648-x)

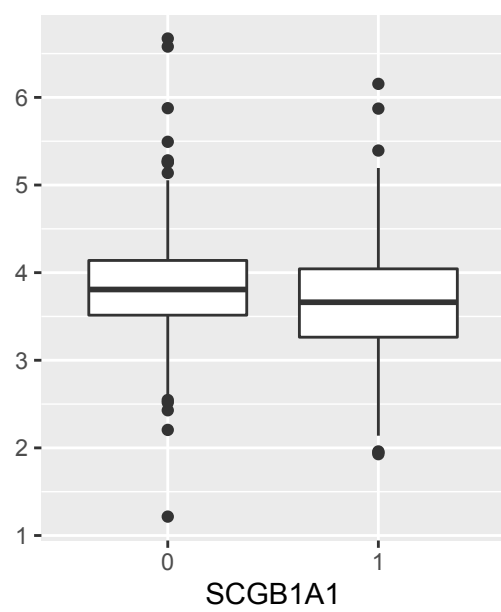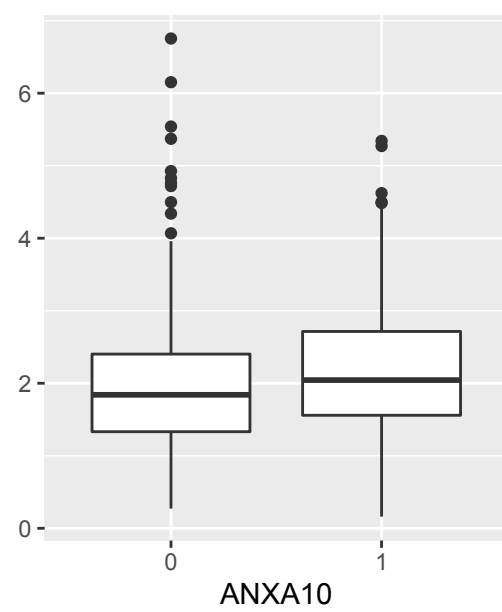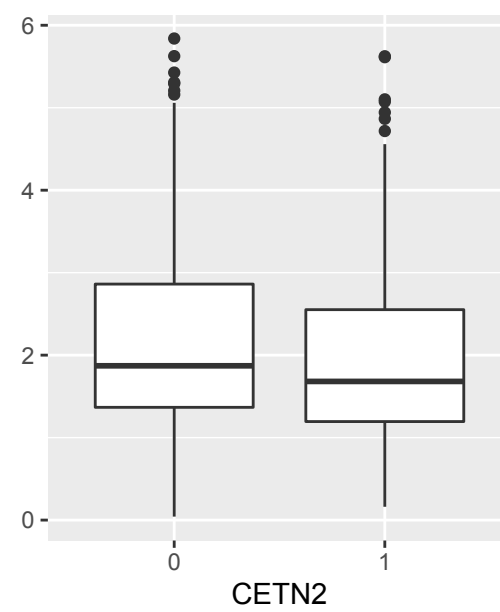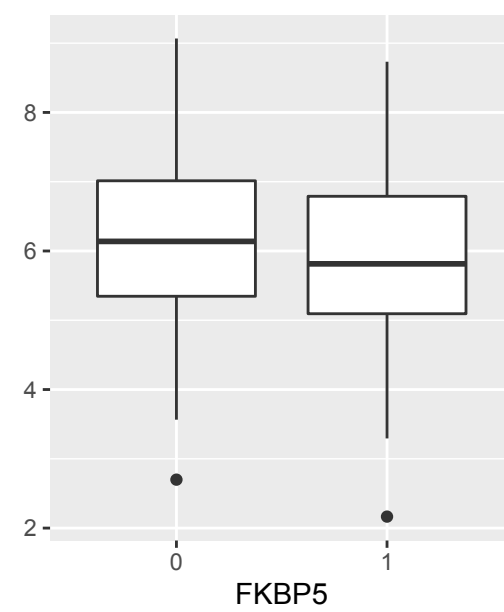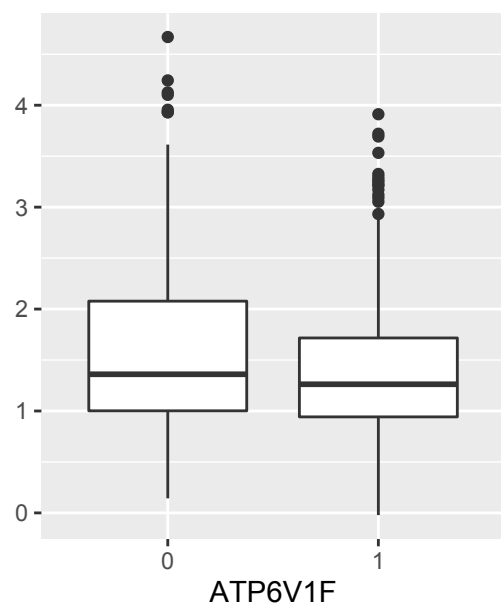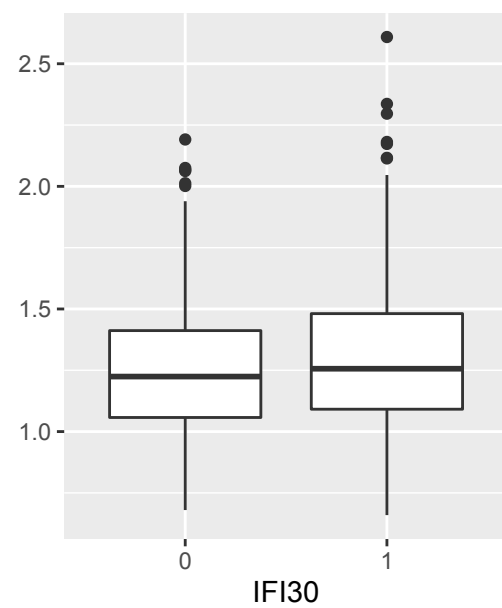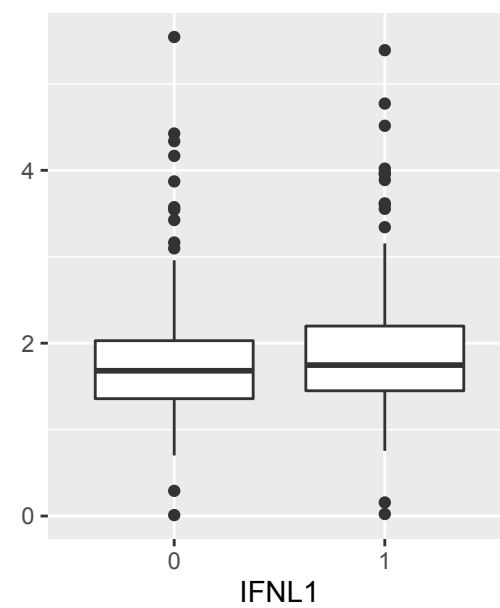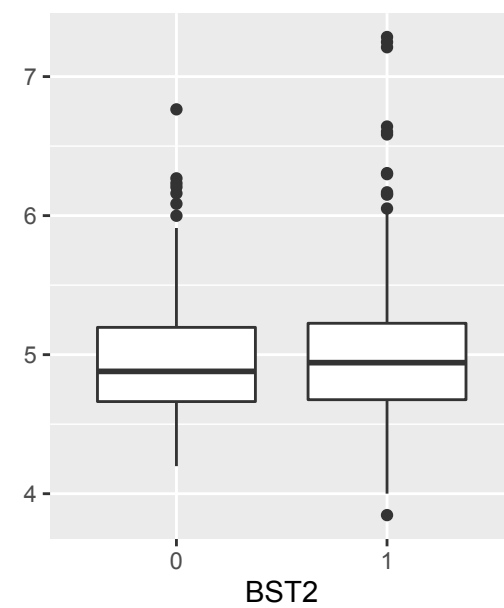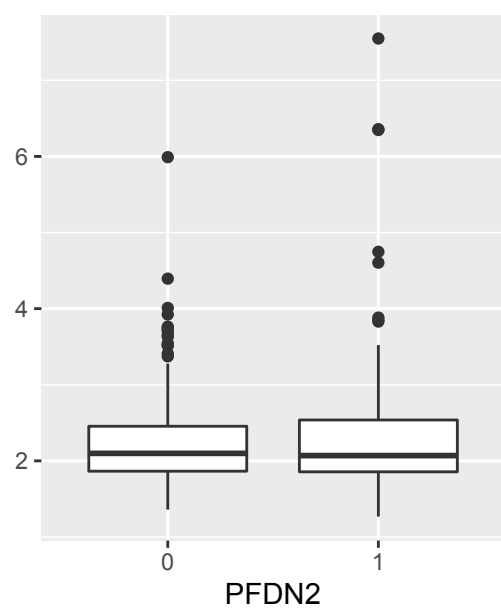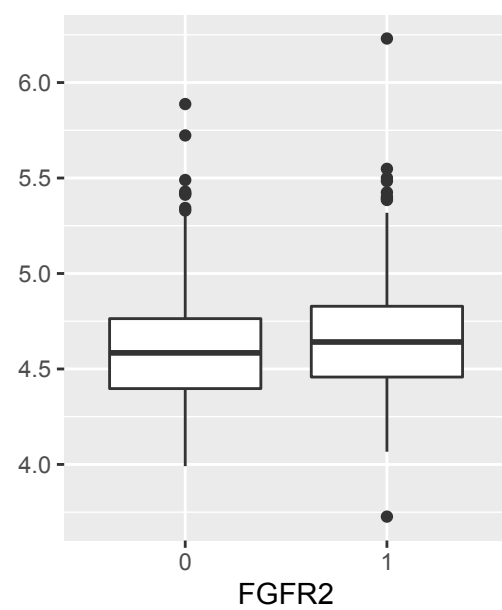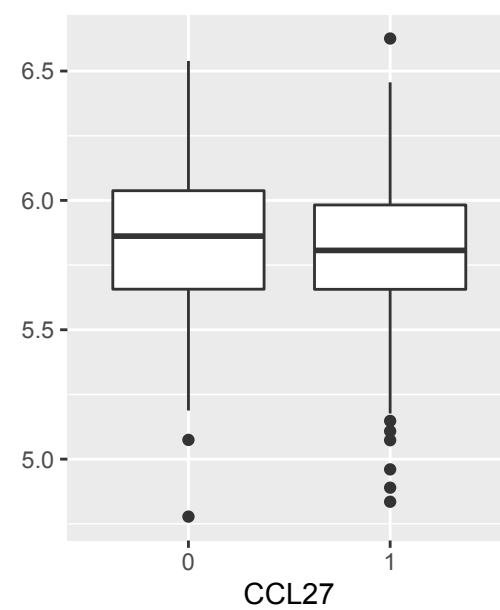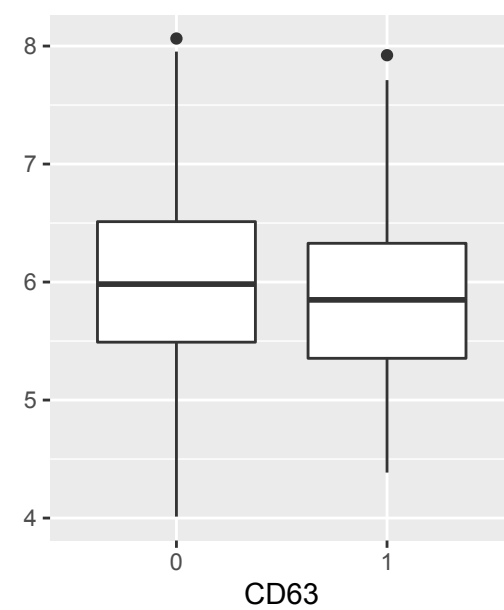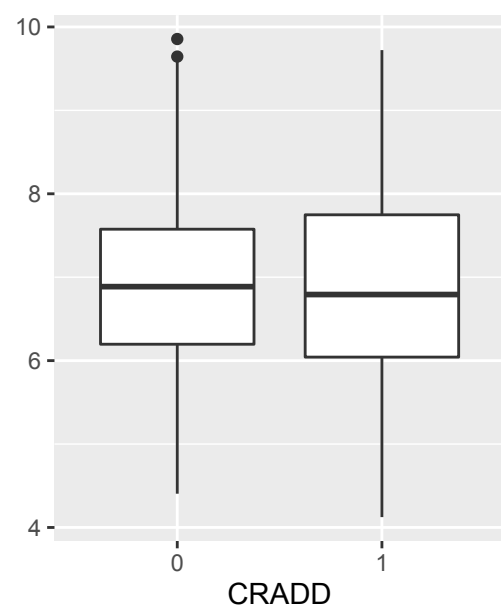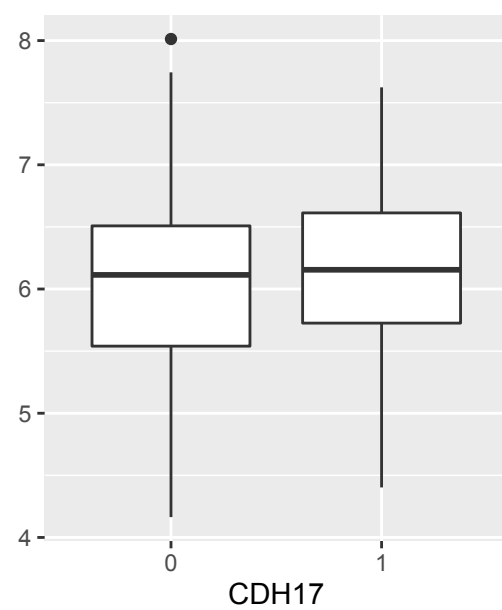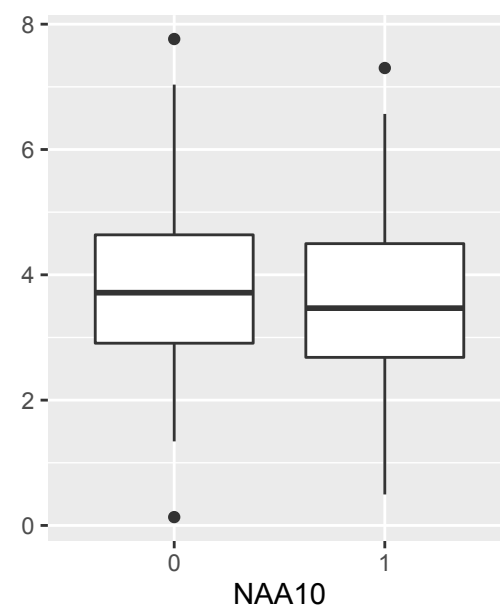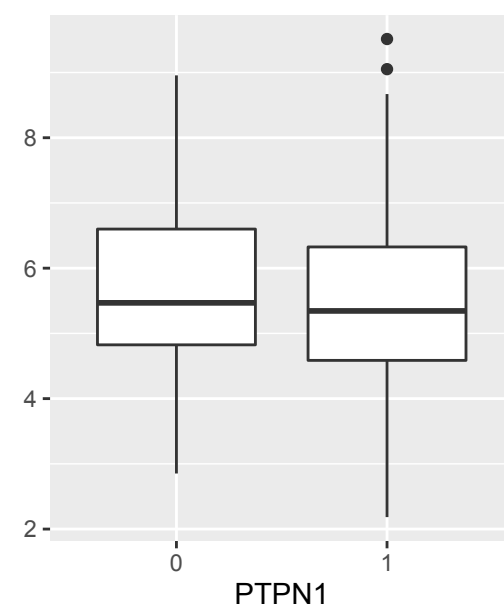

Supplement: Supplementary file 2 — Supplementary Figure 1 [file 41380_2022_1648_MOESM2_ESM.pdf]
